# Bioelectrical Impedance Vector Analysis in Extremely Low-Birth-Weight Infants to Assess Nutritional Status: Breakthroughs and Insights

**DOI:** 10.3390/nu16244348

**Published:** 2024-12-17

**Authors:** Raquel Núñez-Ramos, Diana Escuder-Vieco, Carolina Rico Cruz, Cristina De Diego-Poncela, Sara Vázquez-Román, Marta Germán-Díaz, Nadia Raquel García-Lara, Carmen Pallás-Alonso

**Affiliations:** 1 Department of Pediatrics, 12 de Octubre University Hospital, 28041 Madrid, Spain; 2 Aladina-MGU-Regional Human Milk Bank, 12 de Octubre University Hospital, 28041 Madrid, Spain; diana.e.vieco@gmail.com (D.E.-V.); carolinaricocruz@yahoo.es (C.R.C.); cristinadediegoponcela@hotmail.com (C.D.D.-P.); saravazquezroman@gmail.com (S.V.-R.); nadiaraquelg.nrgl@gmail.com (N.R.G.-L.); kpallas.hdoc@gmail.com (C.P.-A.); 3 Research Institute i+12, 12 de Octubre University Hospital, 28041 Madrid, Spain; 4 Department of Neonatology, 12 de Octubre University Hospital, 28041 Madrid, Spain; 5 Department of Pediatric Nutrition, 12 de Octubre University Hospital, 28041 Madrid, Spain; martagermandiaz@gmail.com

**Keywords:** preterm birth, impedance analysis, phase angle, term-corrected age, body composition, morphofunctional assessment

## Abstract

**Background/Objectives**: To obtain bioelectrical data to assess nutritional status for extremely low-birth-weight (ELBW) infants upon reaching term-corrected age. **Methods**: A descriptive, observational, prospective, and single-center study, which included ELBW preterm infants was performed. The study variables collected were gestational age, sex, and anthropometry at birth and at term-corrected age. Bioelectrical impedance vector analysis (BIVA) was performed by a phase-sensitive device (BIA 101 BIVA PRO AKERN srl, Pisa, Italy). The components of the impedance vector—resistance (R) and reactance (Xc)—were normalized for body height (H). For each subject, the measurement was taken between the 36th and 44th weeks of postmenstrual age (PMA). A semi-quantitative analysis of body composition was performed using the vector modality of the BIA. Using the RXc graph method, the bivariate 95% confidence intervals of the mean vectors were constructed. From the bivariate normal distribution of R/H and Xc/H, the bivariate 95%, 75%, and 50% tolerance intervals for this cohort were drawn. The individual impedance vectors were compared with the distribution of the vectors from other populations. **Results**: 85 ELBW infants (40 male, 45 female) were included, with a mean gestational age at birth of 26 + 6 weeks (±1.76). Mean R/H was 870.33 (±143.21) Ohm/m and Xc/H was 86.84 (±19.05) Ohm/m. We found differences in the bioelectrical data with regard to gender, with resistance values being significantly higher in females. Our ellipses align closely with those from other term neonatal cohorts. **Conclusions**: Bioelectrical data and the confidence and tolerance ellipses of an ELBW infant cohort are presented and can be used as a reference standard for nutritional assessment at discharge.

## 1. Introduction

The evolution of neonatal care units over the last two decades has led to a significant improvement in survival rates for children born at increasingly earlier gestational ages with lower birth weights [1]. Among these infants, those with ELBW (weight at birth of less than 1000 g) stand out for their particular complexity and vulnerability: their growth rate is accelerated, energy stores are low, and functional immaturity inhibits their ability to adapt to an unbalanced supply of nutrients, whether in excess or deficiency [2]. For this reason, they are considered patients at high risk for nutrition-related complications, and their growth often deviates from what is expected. Feeding is a fundamental aspect of their care while in neonatal intensive care, and not only impacts their weight but also their psychomotor development, making it a major challenge for neonatologists [3]. In this context, nutritional intervention is designed to promote adequate catch-up growth, taking into account gestational age, prior history of intrauterine growth restriction, or the degree of postnatal malnutrition, which often affects these patients during hospitalization [4]. In clinical practice, catch-up growth is achieved when a patient reaches the 10th percentile in weight. Nevertheless, this anthropometric measurement, while the most sensitive parameter in the short term, is limited in its ability to provide comprehensive nutritional information and is often altered by the state of hydration.

In this context, it becomes increasingly important to assess body composition (BC). In utero, from weeks 24–27, numerous changes occur, which must be understood in order to guide the nutritional support of premature infants: body mass quadruples, body water as a percentage of body weight decreases, and the proportion of both lean body mass and fat mass progressively increases [5,6].

Previous observational studies using dual-energy X-ray absorptiometry (DXA), air-displacement plethysmography (ADP), and magnetic resonance imaging (MRI), have shown that when a very preterm infant reaches term-corrected age, they have a higher proportion of fat mass and a lower proportion of lean mass compared to those infants born at term [7,8]. Additionally, these differences in BC can lead to increased metabolic risk as an adult. Some of the risk factors associated with metabolic syndrome, such as arterial hypertension, dyslipidemia, impaired glucose tolerance, or increased insulin resistance, correlate inversely with birth weight and appear more frequently when there is rapid postnatal weight gain [9]. However, for reasons not well understood, we know these possible changes in premature infants’ BC are not always sustained in the long term [10].

Over the last few years, bioelectrical impedance analysis (BIA) has become a widely used method to study BC in a simple, inexpensive, mobile, non-invasive, and reproducible manner that does not require patient cooperation. It is grounded in the fact that different components of the human body show different resistance (impedance) to electrical currents. Lean muscle mass, which contains most of the body’s fluids and electrolytes, is a good conductor of electricity (low impedance), while fat mass acts as an insulator (high impedance). BIA analyzers introduce a low-frequency alternating current to record impedance (Z) and its two components: resistance (R) and reactance (Xc). R is the simple and constant resistance that the tissue presents to the flow of current, while Xc reflects the delay in the current flow, indicative of the tissue’s ability to store energy. In short, R is inversely correlated with the hydration and electrolyte content of a tissue and Xc is related to the cell size and membrane integrity. In addition to R and Xc, BIA provides a third parameter, phase angle (Pha), a measurement obtained from the ratio of reactance to resistance (Pha = arctangent (Xc/R) × 180°/π), independent of conventional regression equations for estimating body composition. Some authors advocate that this parameter could be a more sensitive tool for evaluating nutritional status due to its relationship with body cell mass [11], and it has been suggested as a predictor of mobility and mortality in various clinical situations [12,13,14].

BIA results can be assessed quantitatively (conventional BIA) or qualitatively (bioelectrical impedance vector analysis, BIVA). Conventional analysis uses predictive equations that turn raw bioelectrical values into BC variables in order to estimate hydric volumes. To do so, it is necessary to use models that have been developed and validated in populations similar to the study group. Although valid models have been established for adults and older children, conventional BIA relies on a series of assumptions about density, hydration, and geometric proportions that do not hold true in the newborn population, leading to potentially serious errors if applied to newborn populations, especially in the case of premature infants.

These difficulties are circumvented in BIVA, a type of analysis developed by Piccoli et al. [15], which does not depend on predictive models or equations as it uses direct vector impedance measurements that are then compared with data obtained from a reference population through RXc graphs. This reference graph is made up of three concentric ellipses corresponding to the 50th, 75th, and 95th percentiles of the normal distribution of the impedance vector of the reference population. BIVA is a highly attractive strategy for clinical practice in neonatal populations. Following the initial Piccoli et al. study [16], which included 163 subjects (87 males and 75 females) with postnatal ages of 1 to 7 days, other authors have successfully applied this technique to newborns and young infants [17,18,19]. The impedance vector distribution in neonates was determined in Spain by Redondo del Rio et al. [20]. These authors evaluated a group of 154 healthy term newborns (79 males and 75 females) aged 24 to 72 h, showing lower values of resistance and slightly higher reactance values compared to Italian newborns in the first 7 days from the Picolli et al. group. This study proposed tolerance ellipses for Spanish term neonates. However, few studies have examined the preterm population, and bioimpedance analysis is limited. Our goal is to offer the first bioelectrical data for ELBW infants upon reaching term-corrected age.

## 2. Materials and Methods

### 2.1. Study Design and Subjects

A descriptive, observational, prospective, single-center study included ELBW preterm infants born between July 2021 and February 2024. The study was carried out in the level III neonatal unit of 12 de Octubre University Hospital in Madrid, Spain, a reference center for the care of premature infants. Inclusion criteria were as follows: ELBW in a stable condition at the time of pre-discharge or at the time of the first follow-up appointment (between 36th and 44th weeks of PMA) whose parents agreed to participate in the study by signing an informed consent form. The study was conducted in accordance with the Declaration of Helsinki, and the protocol was approved by the Ethics Committee of 12 de Octubre University Hospital Research Institute (Project identification code 21/154; date of acceptance 13 April 2021). The following data were collected: gestational age, sex, and anthropometry (weight and height) at birth and at the time of examination with the corresponding Z-scores using Fenton’s growth charts [21] up to 44 weeks of PMA. Finally, the weight-gain velocity from birth to the time of examination was estimated using Patel’s formula in grams (g) per kilogram per day [22]. Patients were classified according to their obstetric history and anthropometry, depending on the presence or absence of intrauterine growth restriction (IUGR), small for gestational age (SGA), and extrauterine growth restriction (EGR). IUGR was defined as the estimated fetal weight at the time of ultrasound below the 3rd percentile for gestational age and sex, regardless of the presence of Doppler hemodynamic changes, or estimated fetal weight at the time of ultrasound between the 3rd and 10th percentile for gestational age and fetal sex when associated with one or more of the following Doppler hemodynamic changes: umbilical artery pulsatility index (PI) above the 95th percentile for gestational age and/or mean uterine artery PI above the 95th percentile and/or middle cerebral artery PI or cerebroplacental ratio below the 5th percentile for gestational age [23]. SGA was defined as a sex-adjusted birth weight under the tenth percentile (≤1.28 Z-score). EUGR at discharge from the neonatal unit was defined as the difference between Z-scores at birth and discharge with <−2 SD representing severe and −2 to −1 SD representing moderate using Fenton’s growth charts.

### 2.2. Bioelectrical Impedance Vector Analysis

**Figure 1:**
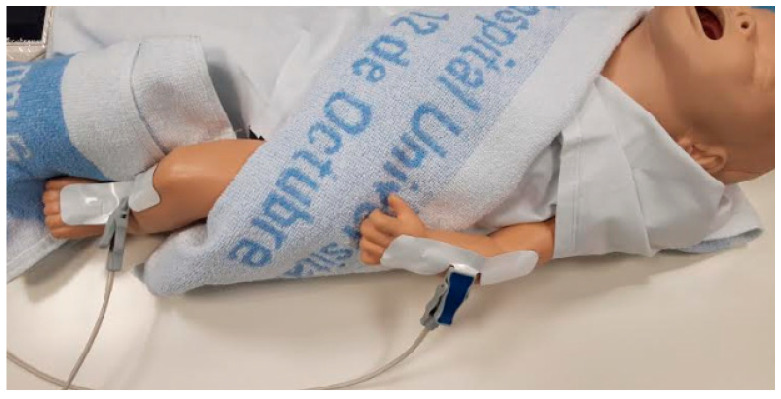
Placement of the electrodes on the patient.

Bioimpedance analysis was performed by a phase-sensitive device (BIA 101 BIVA PRO AKERN srl, Pisa, Italy) working with an alternating sinusoidal electric current of 245 microamperes at an operating frequency of 50 kHz (±1%). The device was calibrated every morning using the standard control circuit supplied by the manufacturer with a known impedance [resistance (R) = 380 ohm; reactance (Xc) = 45 ohm]. The accuracy of the device was 0.1% for R and 0.1% for Xc. The components of the impedance vector—R and Xc—were normalized for body height (H). For each subject, a bioelectrical evaluation was performed between the 36th and 44th weeks of PMA. Three consecutive measurements were taken for each patient at the same moment, with the result being the average (±standard deviation) calculated after verifying a normal distribution. BIA analysis was carried out on patients at the baseline condition, preferably before feeding. Patients were placed in a supine position, undressed, on a non-conductive surface (crib mattress or examination table), ensuring no contact with any metallic surface. The skin that would be in contact with the electrodes was previously cleaned with a 2% chlorhexidine solution. The arms were extended and placed slightly away from the body, and it was ensured that the ankles and thighs were not touching each other (occasionally, a towel or sheet was used to separate the lower limbs). An effort was made to keep the patient calm or even asleep while measurements were being taken, and physical contact between the patient and family members or hospital staff was avoided. Two sets of adhesive Ag/AgCl low-impedance electrodes (BIVATRODES Akern Srl; Florence, Italy), designed for accurate and sensitive bioimpedance measurements, were placed preferably on the hand (blue electrodes) and right foot (gray electrode, Figure 1). In patients with intravenous lines or other local pathologies (burns, lymphedema), electrodes were placed on the upper and lower limbs free of devices and/or lesions. If the patient was receiving intravenous treatment, the infusion was stopped when possible while measurements were taken. Before and after each measurement, the electrodes were cleaned with 70% alcohol. In cases in which the patient was in isolation, the equipment was placed in a sterile plastic bag. Three measurements were taken, preferably by a single researcher, with the result being the average of the bioelectrical parameters. The electrodes were allowed to remain in place for up to 5 h, although the measure was taken within 2–3 min for most patients. Niltac^®^ spray or liquid Vaseline was used to remove the electrodes after measurement.

### 2.3. Statistical Analyses

A semi-quantitative analysis of body composition was performed using the vector modality of the BIA; that is to say that the components of the impedance vector (R and Xc) were normalized by the length of the neonates (R/H [ohm/m] and Xc/H [ohm/m]). Using the RXc graph method, the bivariate 95% confidence intervals (ellipses) of the mean vectors were constructed. From the bivariate normal distribution of R/H and Xc/H, the bivariate 95%, 75%, and 50% tolerance intervals for this cohort of ELBW infants were drawn. Furthermore, the individual impedance vectors were compared with the distribution of the vectors of other populations, including the reference of Piccoli [15] and a Spanish healthy term newborns cohort by Redondo del Río [20] (ellipses of tolerance at 50%, 75%, and 95%). Quantitative variables were described using the mean and standard deviation (SD). Qualitative variables were described using absolute and relative frequencies. Inferencing between populations for bioelectrical data was performed using Student’s t-test, ANOVA, the chi-square (χ^2^) test, or Fisher’s exact test when the number of subjects in several categories was less than 5, based on the nature of the variables. Spearman’s correlation was used to test the association between R/H and Xc/H. All the analyses were performed using SAS 9.4 with a significance level of 5%. The normal distribution of bioelectrical data was evaluated using the Shapiro–Wilk test. The two-sample Hotelling T-squared test was used to compare the differences in the mean bioelectrical impedance vector between the reference values provided by Piccoli et al. [15].

## 3. Results

In total, 85 ELBW infants (40 male, 45 female) were included, with a mean gestational age at birth of 26 + 6 (±1.76) weeks, a mean birth weight of 756.20 (±164.75) g, and a weight z-score of −0.75 (±0.85). The average length of stay in the neonatal care unit was 90.82 (±24.38) days, and the mean weight upon discharge was 2532.47 (±484.79) g, with a weight z-score of −2.10 (±1.11). The mean impedance vector values were obtained for the whole sample prior to discharge or at the first follow-up visit. BIA was carried out with a mean-corrected age of 39 + 1 weeks (±2.12). At the time of measurement, the mean weight and length were 2402.28 (±458.79) g and 43.68 (±2.55) cm, respectively. The results of the bioelectrical data normalized for height (R/H and Xc/H) for the entire sample, categorized by gender, are shown in Table 1.

**Figure 2:**
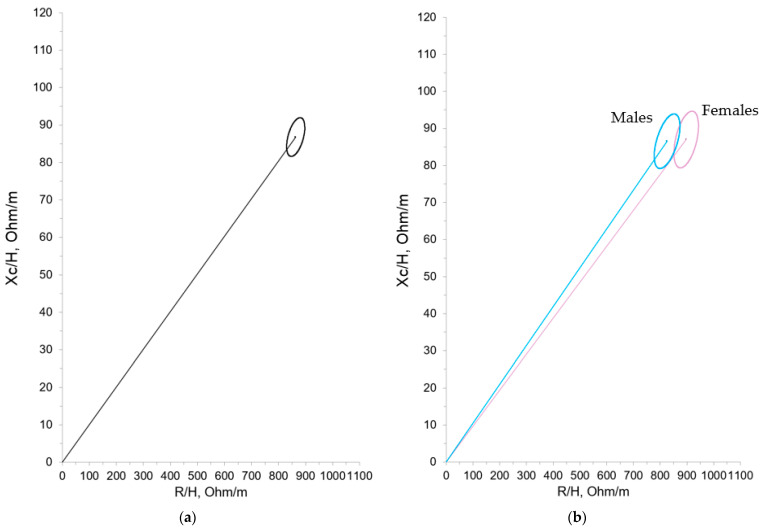
Mean impedance vector with the 95% confidence ellipse for ELBW infants at term-corrected age. (**a**) Total cohort. (**b**) By gender.

Statistically significant differences were found according to gender for the raw R, supporting the construction of both total and separate (males and females) 95% confidence ellipses of the mean impedance vector (Figure 2a,b).

**Figure 3:**
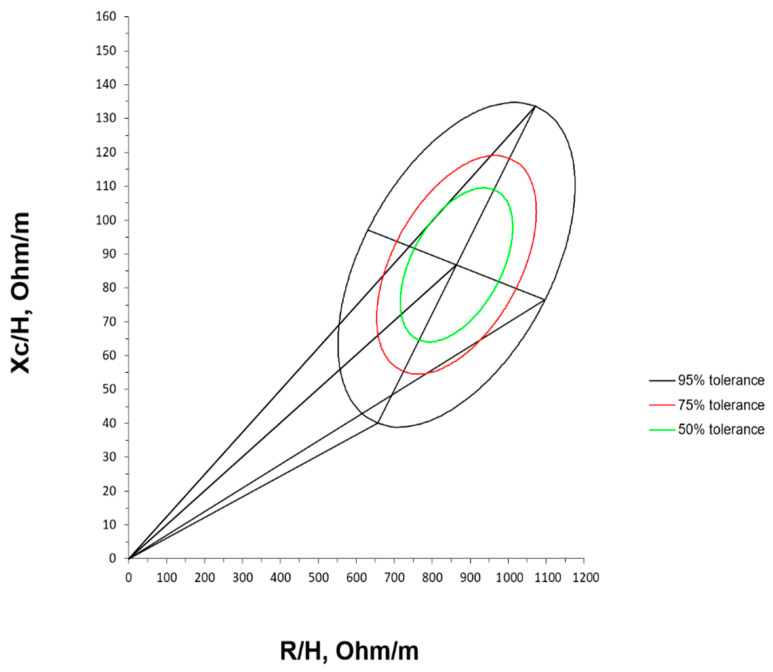
Tolerance ellipses for extremely premature infants at term-corrected age.

The mean impedance vector with the reference 50%, 75%, and 95% tolerance ellipses for extremely premature infants with birth weight < 1000 g at term-corrected age is represented in Figure 3.

**Figure 4:**
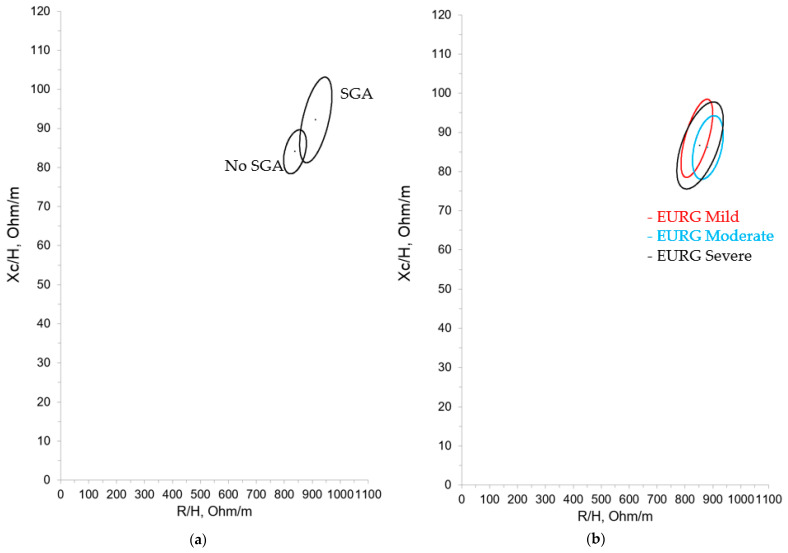
Mean impedance vector with the 95% confidence ellipse for ELBW infants at term-corrected age. (**a**) According to SGA and (**b**) according to EUGR.

**Figure 5:**
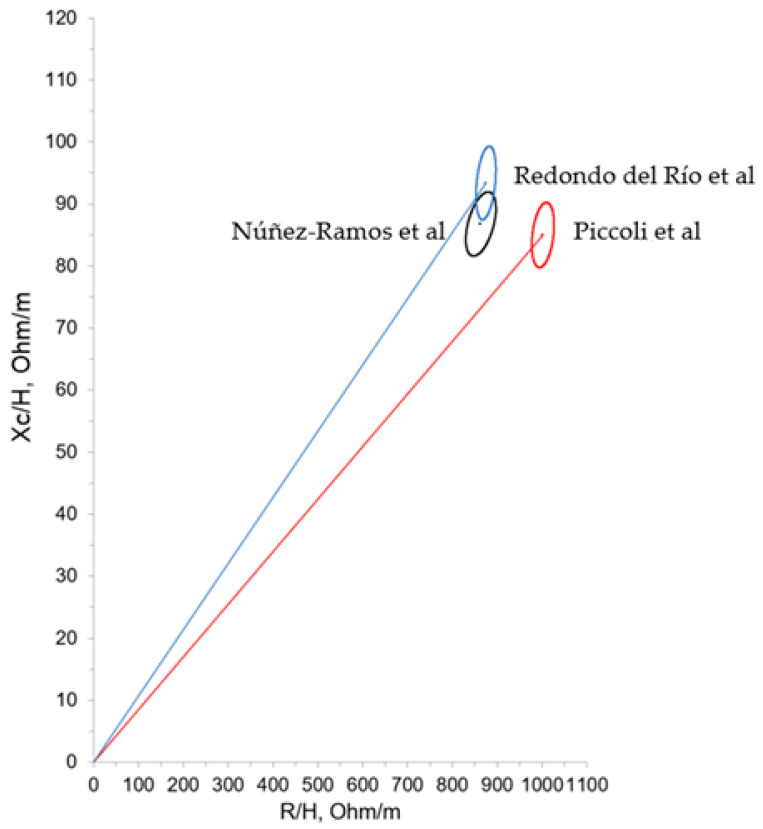
Mean impedance vector with the 95% confidence ellipse for our cohort and Redondo del Río et al.’s [20] and Piccoli et al.’s [16] populations.

Subsequently, the patients included in the study were classified according to different intra and extrauterine growth patterns. Forty percent of patients had a history of IUGR and 32.94% were SGA. Moreover, 43.53% met the criteria for moderate EGR and 20% for severe EGR, with an average growth velocity during hospitalization of 12.11 (±1.66) g/Kg/day. Table 2 and Table 3 summarize the bioelectrical data for these categories. Figure 4 and Figure 5 show the confidence ellipses based on the criteria of SGA and mild, moderate, or severe EUGR.

Data from our cohort were compared with the bioelectrical results of healthy neonates in the first 7 days from Piccoli et al. [16] and healthy neonates in the first 3 days from Redondo del Río et al. [20] (Figure 5).

**Figure 6:**
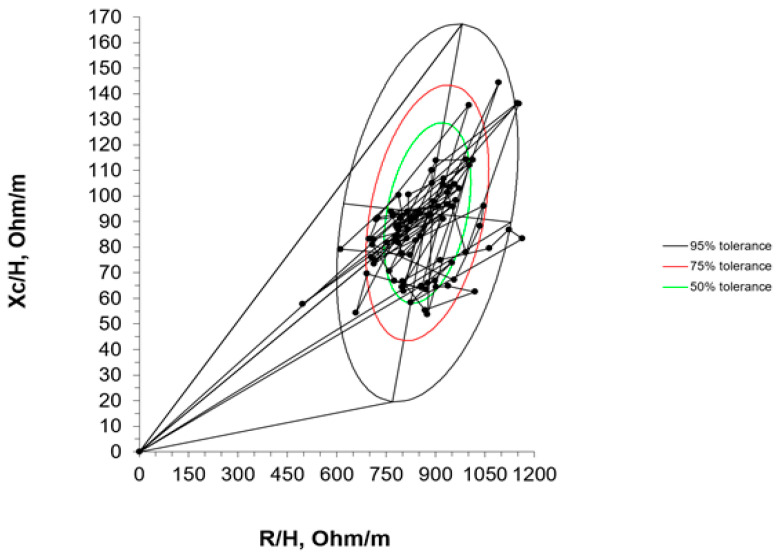
Individual impedance vectors in Redondo et al.’s [20] ellipses.

Finally, the comparison of individual vectors of the evaluated ELBW infants at term-corrected age with the reference tolerance ellipses of the study performed by Redondo del Río et al. [20] is shown in Figure 6.

## 4. Discussion

This study describes, for the first time, bioelectrical data for ELBW preterm infants at term-corrected age. Additionally, the confidence and tolerance ellipses are constructed, which can be used as a reference pattern for the nutritional evaluation of this population.

For ELBW infants, there is increasing evidence that growth during admission to neonatal units has an impact on their overall outcome. However, there is much controversy about the definition of optimal growth in this population of newborns and what the best parameters to monitor it or to achieve this adequate growth are [24,25]. Although nutritional recommendations for preterm infants are under constant review and discussion, they generally use fetal growth rates as a reference point, an objective that oftentimes is not met before hospital discharge or at the age of term, leading almost unavoidably to malnutrition, especially in those infants with lower gestational age and weight. Furthermore, even when anthropometric values are similar, the clinical situation can vary greatly from one patient to the next. For this reason, BIA is being used in clinical practice, even in the newborn population, in order to evaluate nutritional status more thoroughly. BIA is rapid, reproducible, non-invasive, and safe. However, there are few studies focusing on the preterm infant population, BC data obtained by BIA is scarce, and in the case of ELBW, these data are practically non-existent. To date, only a few isolated studies have been published in which subjects have higher gestational ages and average weight [26,27].

To the best of our knowledge, the data we present belong to the largest newborn cohort of ELBW infants ever reported, in which the data were obtained at term-corrected age and using the vector modality of the BIA (BIVA). For the first time, confidence and tolerance ellipses for ex-preterm infants weighing less than 1000 g at the time of birth are presented. These data could be used as a reference pattern for the nutritional evaluation of this collective at the time of discharge.

We compared our results with those obtained by Redondo del Río et al. [20], who evaluated a group of 154 healthy Spanish newborns (79 males, 75 females) aged 24 to 72 h, with a gestational age greater than or equal to 37 weeks (37–41 weeks) and an appropriate weight for gestational age. Just as in Redondo del Rio et al. [20], we found differences in the bioelectrical data with regard to gender, with resistance values being significantly higher in females. However, these differences have not been confirmed in other studies of term neonates and young infants; consequently, their clinical significance cannot be precisely established, nor can we say with certainty that different reference ellipses must be used with this population. Still, based on our experience, it could be beneficial, as shown in Figure 2b where the ellipses show minimal overlap. On the other hand, in the different growth patterns studied (IUGR, SGA, and EGR), we only found statistically significant differences in the case of the SGA population. It can be observed that the SGA group showed higher R/H and Xc/H values, which suggests decreased hydration and an increase in total cell mass. The IGR group showed the same tendency (higher R/H and Xc/H values with respect to non-IGR patients) although the differences did not reach statistical significance. Considering the size of these subgroups, it could be imprecise to raise hypotheses, but these results may show an intervention bias in favor of SGA and IGR populations at birth, with more early and aggressive nutritional interventions. In contrast, the bioelectrical data in the different EGR categories (mild, moderate, and severe) were very similar, and therefore, unsurprisingly, their confidence ellipses showed considerable overlap.

Our ELBW infant group was compared with healthy neonates in the first 7 days from Piccoli et al. [16] and healthy neonates in the first 3 days from Redondo del Rio et al. [20]. In both populations, newborns were born at term. The values obtained were similar to those found in these studies, as can be visualized by the confidence ellipses of the three groups (Figure 6), which implies that it is technically feasible to perform BIVA in the population of ELBW infants at term age.

Our ellipses align most closely with those from the Redondo del Rio et al. [20] cohort. For this reason, individual impedance vectors of our ELBW infants were represented in the ellipses from this group. Considering body composition according to vector placement, most of our patients were located in the lower right and left quadrant (known for their association with cachectic and obese sections in adult patients) as they had, on average, lower values of reactance (Xc/H = 86.84 Ohm/m in our study, Xc/H = 93.4 Ohm/m in that of Redondo del Rio et al. [20]). Considering term newborns as a reference population, our data therefore showed a lower vector length, suggesting more fluids than the term neonates, possibly due to inflammation or a worse cellular quality.

As a primary limitation, the sample sizes of patient subgroups according to their intra- and extrauterine growth patterns may not have been large enough to detect differences in the parameters studied here. This is a single-center study targeting an extraordinarily vulnerable population with high mortality and morbidity, making it difficult to recruit participants. Although all the phase-angle values were similar across all the analyzed subgroups, included in all categories of EGR, the design of our study did not allow for an analysis of the prognostic value of BIVA in this context. Although just over half of the investigated ELBW preterm infants had a mother of Spanish origin, it was a multicultural sample in which comparisons between populations groups were not considered to be representative, and the impact of ethnic origin in BIVA results was not addressed. Future studies focusing on the impact of extrauterine growth and racial populations on bioelectrical impedance are needed.

Bioelectrical impedance in newborns is a growing field with significant potential for clinical nutrition research. Studying BC in this critical stage of development can help to discover the characteristics of catch-up growth, personalize nutritional interventions for this population, and improve their long-term health outcomes.

## 5. Conclusions

The study of bioelectrical parameters in the ELBW population is feasible and can be of great value to guide nutritional management. The confidence and tolerance ellipses of a broad ELBW infant cohort at term-corrected age are presented, which can be used as a reference standard for nutritional assessment at discharge.

## Figures and Tables

**Table 1:** Bioelectrical data categorized by gender, expressed as mean (SD).

Variable (Mean y SD)	Total Sample (n = 85)	Females (n = 45)	Males (n = 40)	*p* Value
R (Ohm)	379.14 (±59.03)	392.69 (±44.87)	363.90 (±69.17)	0.024
R/H (Ohm/m)	870.33 (±143.21)	897.27 (±120.11)	840.01 (±161.61)	0.065
Xc (Ohm)	37.78 (±7.82)	38.02 (±8.09)	37.51 (±7.60)	0.77
Xc/H (Ohm/m)	86.84 (±19.05)	87.00 (±20.02)	86.65 (±18.15)	0.93
Phase angle	5.65 (±1.09)	5.44 (±1.09)	5.88 (±1.05)	0.061
r	0.4145 *	0.4013 *	0.4227 *	

r, linear correlation coefficient between R/H and Xc/H. * *p* < 0.05.

**Table 2:** Bioelectrical data categorized by gender, expressed as mean (SD).

Variable (Mean and SD)	Total Sample (n = 85)	IUGR			SGA		
		Yes (n = 34)	No (n = 51)	*p* Value	Yes (n = 28)	No (n = 57)	*p* Value
R/H (Ohm/m)	870.33 (±143.21)	901.18 (±180.71)	849.75 (±180.74)	0.11	938.30 (±164.75)	836.93 (±119.31)	0.002
Xc/H (Ohm/m)	86.84 (±19.05)	88.35 (±22.68)	85.83 (±16.37)	0.55	92.26 (±22.86)	84.17 (±16.45)	0.065
Phase angle	5.65 (±1.09)	5.55 (±1.10)	5.71 (±1.09)	0.51	5.56 (±1.13)	5.69 (±1.08)	0.59
r	0.4145 *	0.5951 *	0.2678		0.5484 *	0.3404 *	

r, linear correlation coefficient between R/H and Xc/H. * *p* < 0.05.

**Table 3:** Bioelectrical data categorized according to EGR, expressed as mean (SD).

Variable (Mean y SD)	EGR			
	Mild (n = 31)	Moderate (n = 37)	Severe (n = 17)	*p* Value
R/H (Ohm/m)	867.93 (±167.77)	879.94 (±132.31)	853.76 (±122.47)	0.82
Xc/H (Ohm/m)	88.37 (±20.48)	85.63 (±19.37)	86.67 (±16.35)	0.84
Phase angle	5.87 (±1.10)	5.49 (±1.18)	5.74 (±0.84)	0.51
r	0.4480 *	0.4144 *	0.2696	

r, linear correlation coefficient between R/H and Xc/H. * *p* < 0.05.

## Data Availability

The research data presented in this study are available upon request from the corresponding author.
